# Orbital Tumors—Clinical, Radiologic and Histopathologic Correlation

**DOI:** 10.3390/diagnostics12102376

**Published:** 2022-09-30

**Authors:** Daniel Vogele, Nico Sollmann, Annika Beck, Benedikt Haggenmüller, Stefan Andreas Schmidt, Bernd Schmitz, Thomas Kapapa, Yigit Ozpeynirci, Meinrad Beer, Christopher Kloth

**Affiliations:** 1 Department of Diagnostic and Interventional Radiology, Ulm University Medical Center, Albert-Einstein-Allee 23, 89081 Ulm, Germany or; 2 Institute of Pathology, Ulm University Medical Center, Albert-Einstein-Allee 23, 89081 Ulm, Germany; 3 Department of Neuroradiology, Bezirkskrankenhaus Günzbrg, Lindenallee 2, 89312 Günzburg, Germany; 4 Department of Neurosurgery, Ulm University Medical Center, Albert-Einstein-Allee 23, 89081 Ulm, Germany; 5 Institute of Neuroradiology, University Hospital, LMU Munich, Marchioninistr. 15, 81377 Munich, Germany

**Keywords:** benign orbital masses, malignant orbital masses, radiological imaging, histopathology

## Abstract

Orbital masses include a broad spectrum of benign and malignant entities. Often these masses are asymptomatic or show a slow growth rate, so that emergence of clinical symptoms is prolonged. In this context, cross-sectional imaging plays an elementary role in the characterization of these lesions. Aside from the characterization of the underlying entity, an evaluation of the involved compartments is possible by sufficient imaging, which also facilitates optimal treatment and surgery planning. The purpose of this review is to explore different benign and malignant orbital tumors and their typical appearance in imaging together with histopathologic findings.

## 1. Introduction

Vision is the most important of the five human senses. Human beings receive more than 80% of their sensory perception through the eyes. Traumatic accidents and tumorous lesions are the most common reasons to perform the dedicated imaging of the orbit [1]. Clinical history and physical examination may be limited or delayed until the exclusion or treatment of more life-threatening conditions has been achieved [1]. However, two thirds of all orbital tumors are benign, but they can lead to a significant reduction of the quality of life with the risk of vision disorder [2,3]. With the early and correct diagnosis of orbital pathologies supported by dedicated imaging, the best treatment, pharmacological or surgical, can be initiated to save patients’ vision and limit complications (e.g., the progressive infiltration or destruction of surrounding structures). For radiologists it is important to know common orbital lesions and their typical patterns and appearance in imaging. In addition to ultrasound, computed tomography (CT) or magnetic resonance imaging (MRI) are typically used for orbital imaging. In particular, the high-resolution MRI of the orbit has become an accepted diagnostic technique for a wide range of orbital diseases and tumors [4,5]. MRI can be combined with surface coils for a better soft-tissue resolution and lesion characterization [6]. Aside from the assignment to a dignity and a specific entity, a detailed description of involved compartments and anatomical structures is important. In this context, the orbital space has four different anatomic compartments: the bulbus/globe, the intraconal and extraconal spaces, and the optic nerve sheath complex [6].

This pictorial review describes the most common tumorous orbital disorders. In addition, the imaging techniques and radiological findings for each pathology are described.

## 2. Anatomy of the Orbit

Detailed knowledge of the anatomy of the orbit is important. The exact description of the anatomical location of the tumors and the involved structures are crucial for diagnosis on the one hand and for further therapy, e.g., surgical treatment, on the other hand [7]. The orbit can be divided in different compartments. The extraocular muscles control eye movements and, except for the obliquus inferior muscle, form the muscle cone. The muscle cone converges at the orbital apex and divides the orbit into the intraconal and extraconal compartments. The intraconal compartment includes the globe, the optic nerve-sheath complex, and orbital vessels and nerves. The extraconal compartment consists of the bony orbital walls, fat, and the lacrimal gland [8].

## 3. Imaging Techniques

This pictorial review is based on cases of orbital tumors collected from the Department of Diagnostic and Interventional Radiology at the Ulm University Medical Center (Ulm, Germany). MRI was performed with a 1.5T or 3.0T system (Siemens Avanto/Skyra, Siemens Healthineers, Erlangen, Germany), whereas CT images were acquired with a 128 slice scanner (Siemens Somatom AS+, Siemens Healthineers, Erlangen, Germany).

The MRI data were acquired with the use of a phased-array surface coil system. The institutional protocol includes T1- and T2-weighted oblique axial images along the plane of the optic nerves, oblique T2-weighted images, and diffusion-weighted imaging. If an intravenous contrast agent was applied, all sequences after application were acquired with fat suppression. The sequence protocol for orbit imaging is shown in Table 1.

### 3.1. Intraocular Tumors

#### 3.1.1. Retinoblastoma

Retinoblastoma is an aggressive eye tumor of children and infants, representing the most common intraocular neoplasm in childhood [9]. Of all patients with retinoblastoma, 95% are diagnosed before the age of five years [10]. About 40% of patients with retinoblastoma have bilateral tumors. In case of bilateral manifestations, an underlying germline mutation must be suspected. However, in unilateral tumors, only around 15% of cases are caused by mutations. According to this, tumors can be subdivided into sporadic and heritable [10].

Despite aggressive growth patterns, the overall survival rate is very high (>95%). Nevertheless, the prognosis of metastasized retinoblastoma remains poor [11]. For retinoblastomas that are detected prior to metastatic spread, different conservative therapies are available including laser coagulation, thermotherapy, cryotherapy, brachytherapy, and chemotherapy. In clinical examinations, patients showed a leukocoria and a loss of red-eye reflex [12].

**Figure 1:**
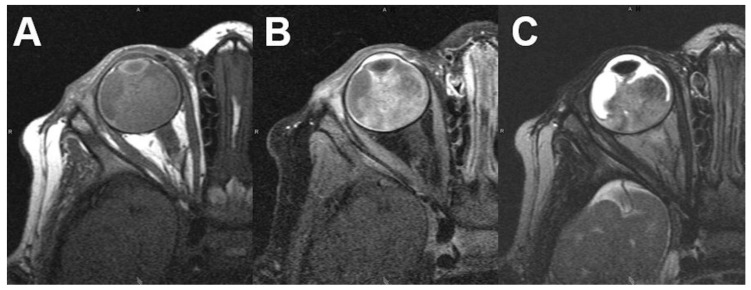
***Retinoblastoma***. MRI of a 16-months-old male patient with right-sided retinoblastoma. T1-weighted images show a slightly hyperintense tumor (**A**) with diffuse contrast enhancement after contrast administration (**B**). On T2-weighted images the tumor presents with a hypointense signal compared to the vitreous body (**C**).

The MRI of the orbit is the standard method for assessing extraocular growth and the possible infiltration of the optic nerve. On T2-weighted images, retinoblastoma is hypointense compared to the vitreous body or cerebrospinal fluid. The calcified parts of the tumor can appear as hypointense foci on both T1- and T2-weighted images. On T1-weighted images, retinoblastoma is slightly hyperintense, with moderate to marked enhancement after the administration of a contrast agent. In addition, retinoblastoma shows restricted diffusion on DWI and low signal in the apparent diffusion coefficient (ADC) maps [13,14]. Both endophytic (into the vitreous body) and exophytic growth patterns could be registered and should be described in the radiological report [15]. The infiltration of the optic nerve is difficult to detect with MRI; however, contrast enhancement is no reliable indicator for involvement [16,17]. Furthermore, a recent study showed that the infiltration of the optic nerve can also be predicted by the contrast enhancement of the anterior chamber [18]. An example of a patient with retinoblastoma is shown in Figure 1.

Different clinical classification systems exist; the most commonly used ones are the International Intraocular Retinoblastoma Classification (IIRC) and Intraocular Classification of Retinoblastoma (ICRB) [19,20]. An overview of Intraocular Classification of Retinoblastoma (ICRB) is given in Table 2.

The involvement of the optic nerve is associated with a poor prognosis, with a mortality rate ranging from 13% to 89%, depending on the degree of optic nerve involvement and whether the surgical margin is clear [10]. In this context, optic nerve involvement is generally indicated by the interruption of a linear area of enhancement at the choroidoretinal complex; however, MRI is limited for this point with an accuracy rate of 52% to 79% [10]. In patients with bilateral disease, also an intracranial tumor is possible called trilateral retinoblastoma, which is present in approximately 5% to 7% of all bilateral cases [21]. The most common locations are the midline, suprasellar, or pineal region. For this reason, the pineal region should be included in the orbital imaging when retinoblastoma is suspected.

#### 3.1.2. Uveal Melanoma

Uveal melanoma is the most common primary intraocular malignancy in adults [22]. It represents 85% of ocular melanomas. Remaining ocular melanomas originate from the conjunctiva (5%) or other sites (10%) [23]. In contrast to cutaneous melanoma, which arises from melanocytes of the basal layer of the epidermis, uveal melanomas arise from melanocytes in the uveal tract. Located in the choroid are 85 to 90%, while a minority is located in the iris or ciliary body. However, large tumors often involve multiple parts of the uvea [24]. Due to the lack of lymphatic channels in the uveal tract, metastasis only occurs through local extension or blood dissemination. Up to 50% of the patients develop metastases, in which the liver is the most common manifestation site with a fraction of about 90% [25]. The optimal treatment is mainly determined by the location and size of the tumor.

**Figure 2:**
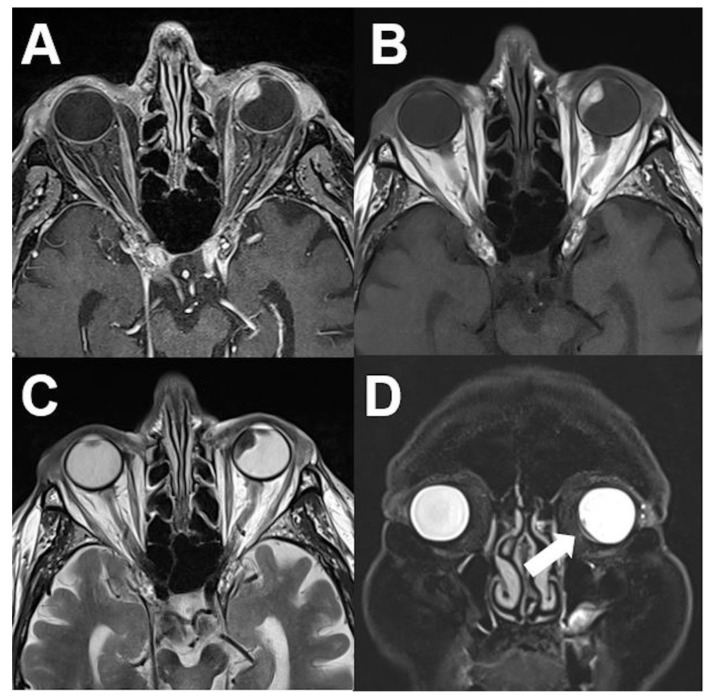
***Uveal melanoma.*** MRI of an 84-year-old male patient with uveal melanoma showing a well-defined tumor of the left nasal vitreous body. The tumor shows a hyperintense signal on unenhanced T1-weighted images (**A**), a diffuse enhancement on contrast-enhanced T1-weighted images (**B**), and a marked hypointense signal on T2-weighted images (**C**). Retinal detachment with subretinal fluid (arrows) is shown in the coronal view (fat saturated T2-weighted sequence) (**D**).

Ultrasound only measures two-dimensional cross sections of the tumor, whereas MRI makes it possible to image the complete tumor in three dimensions and the possible extrascleral extensions. The typical appearance of the uveal melanoma is a well-defined, hyperintense tumor on non-enhanced T1-weighted images compared to the vitreous body. The hyperintense signal depends on the tumors´ melanin content. A moderate contrast enhancement can be detected; nevertheless, it is hardly visible due to the intrinsic T1 hyperintensity. In addition, uveal melanoma often causes retinal detachment and subretinal hemorrhage (Figure 2).

Uveal melanoma can show different morphologic features, such as lentiform, dome, or mushroom shape. Tumor shape itself mirrors the type of tumor development and therefore has a prognostic relevance. Flat lesions may remain small over a long period; however, they can infiltrate the sclera [26,27]. Growing tumors can break through the Bruch’s membrane (extracellular matrix located between the retinal pigment epithelium and the choroid) and extend into the subretinal space with the typical mushroom shape [28]. The mushroom shape is characterized by a stalk with a diameter smaller than that of the summit. On the other hand, dome shaped tumors demonstrate a displacing development [27].

**Figure 3:**
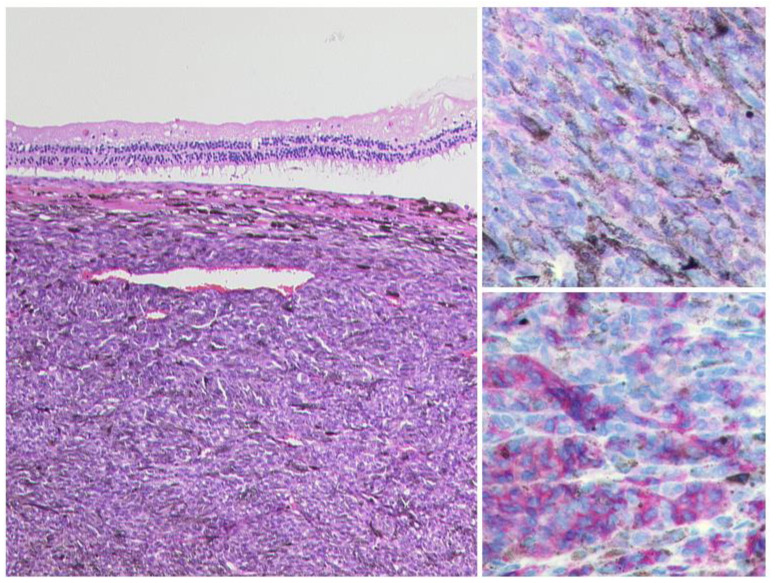
***Uveal Melanoma.*** Left: A pigmented tumour mass is seen beneath the artificially detached retina, HE, Original magnification 50:1. The tumour cells show a weak expression of S100 (upper right, original magnification 200:1) and a modest expression of Melan A (lower right, original magnification 50:1).

On contrast-enhanced T1-weighted images, the tumor shows moderate diffuse enhancement. On T2-weighted images uveal melanomas show marked hypointense signal compared to the vitreous body. In addition, retinal detachment with subretinal fluid is present. Evaluating the tumor in different planes is needed to assess tumor geometry, for accurate measurements, to identify sclera/extrascleral extension, and to plan an adequate surgical resection [28]. Typically, a lentiform or a “mushroom” shape can be found. Chemical shift artefact at the sclera-orbital fat should not be misinterpreted as sclera, which could result in an overestimation of the sclera thickness [28]. The histopathological findings of a uveal melanoma are shown in Figure 3.

### 3.2. Intraconal Tumors

#### 3.2.1. Venous Varices

Orbital venous varices can relate to an intra- and/or extraconal mass but are rather rarely observed. They account for less than 2% of all orbital tumors [29]. They typically occur unilaterally but bilateral manifestation is also possible [30]. Orbital varices are primarily congenital and may stem from dilatation of only a single or, alternatively, multiple venous vessels. Secondary orbital venous varix can develop due to pathologies with increased blood flow, such as arteriovenous malformations or fistulas, with a drain via the orbit [31]. In general, orbital varices can cause transient diplopia and position-dependent exophthalmos. Acute complications are intraorbital hemorrhage and thrombosis, including symptoms, such as deteriorated visual acuity and retro-orbital pain [29,32]. It is characteristic that during intervals of venous hypertension varices expand, while during intervals of low venous pressure varices can collapse. Thus, the imaging appearance of orbital varices can vary, with periods of completely absent findings of an orbital mass in CT or MRI during low venous pressure. Yet, patients affected by transient exophthalmos, for example provoked during the Valsalva manoeuvre or jugular vein compression or occurring spontaneously during coughing or forced expiration, have high suspicion for orbital varices.

**Figure 4:**
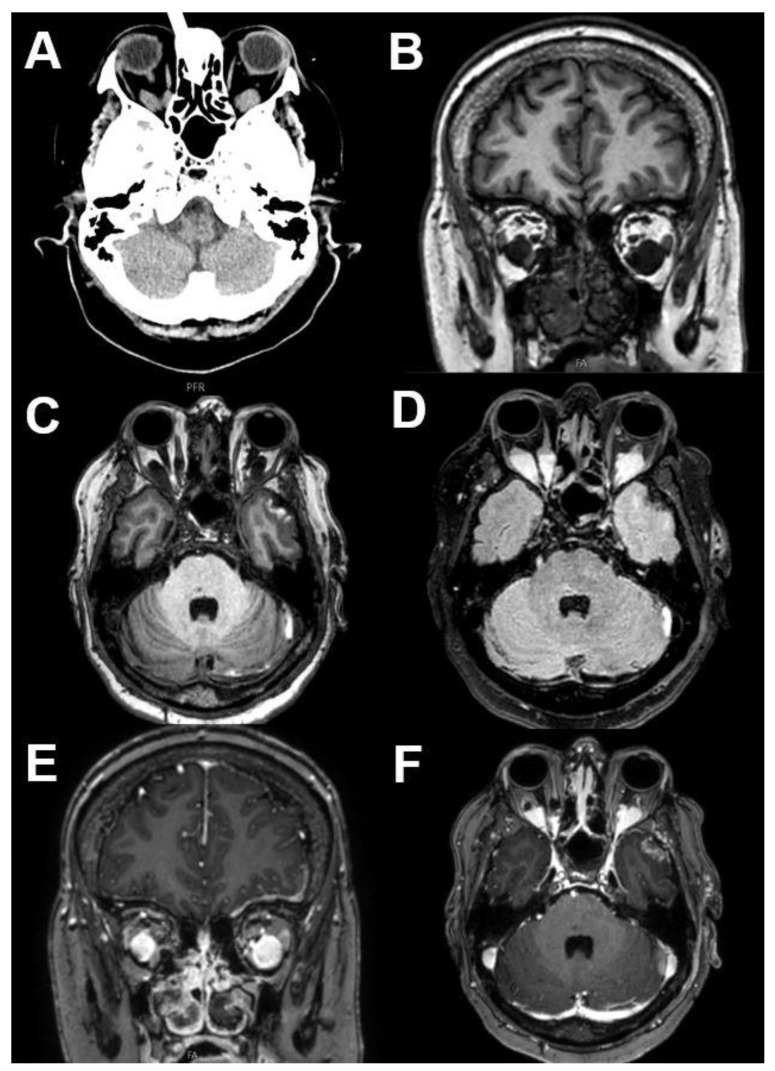
***Venous varices.*** CT and MRI of a patient with bilateral orbital venous varices. CT shows bilateral dorsal orbital mass isodense to intraorbital muscles (**A**). Varices show T1-hypointense (**B**,**C**) and FLAIR-hyperintense signal (**D**). After contrast media application a marked enhancement can be observed (**E**,**F**).

Imaging findings strongly depend on the grade of dilatation of the orbital varices at the time point of image acquisition. Of note, the collapse of varices particularly during image acquisition can be common, given that jugular vein pressure is low during supine position that is, however, standardly used for CT or MRI examinations. Thus, the absence of an orbital mass under these premises does not entirely rule out orbital varices. Consequently, different approaches prior to or during image acquisition have been propagated, including the Valsalva manoeuvre or elastic neck tourniquets to enhance jugular compression [33,34,35]. Furthermore, owing to the lack of valves of the facial venous system, sufficient increase in venous pressure may already be induced by image acquisition in prone position. The typical appearance of orbital venous varix is an intraorbital isodense mass on CT, which shows smooth enhancement that should match that of other venous structures. Regarding MRI, a T1- and T2-hypointense signal and FLAIR-hyperintense signal can be observed, with the marked contrast enhancement of the lesion after contrast media application (Figure 4). If thrombosis is present, a heterogeneous signal can often be observed. The presence of phleboliths, primarily detectable in CT, and a dynamic change in size of the lesion (e.g., the comparison of imaging derived from different time points, comparison between subsequent MRI sequences or provocation by Valsalva manoeuvre) can facilitate correct diagnosis.

#### 3.2.2. Cavernous Hemangioma

Orbital cavernous venous malformation (cavernous hemangioma) is the most common benign ophthalmic tumor in adults. They make up about 5 to 8% of all orbital tumors, mostly present in the middle aged individuals (between 30 and 50 years) [36]. Women are affected more frequently than men. In the case of progression and symptomatic patients (diplopia or visual disturbance), a surgical excision is necessary. Patients present usually with a slowly growing orbital mass resulting in proptosis [37]. Additionally, visual field defects and diplopia are possible.

**Figure 5:**
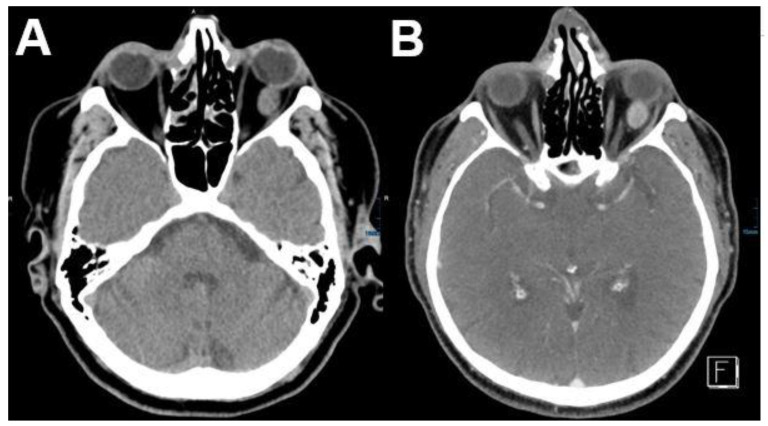
***Cavernous hemangioma.*** CT of a 54-year-old female patient with a left-sided cavernous hemangioma. The tumor appears as an intraconal, well-circumscribed, slightly hyperdense mass (**A**) with mild contrast enhancement (**B**).

Hemangioma is typically surrounded by a fibrous capsule. Overall, cavernous hemangiomas can be located anywhere within the orbit; however, a majority (>80%) are located in the intraconal compartment, especially in the lateral compartment [38]. On CT scans hemangioma presents as a well-defined, homogenous mass with a slightly hyperdense appearance compared to ophthalmic muscles (Figure 5).

**Figure 6:**
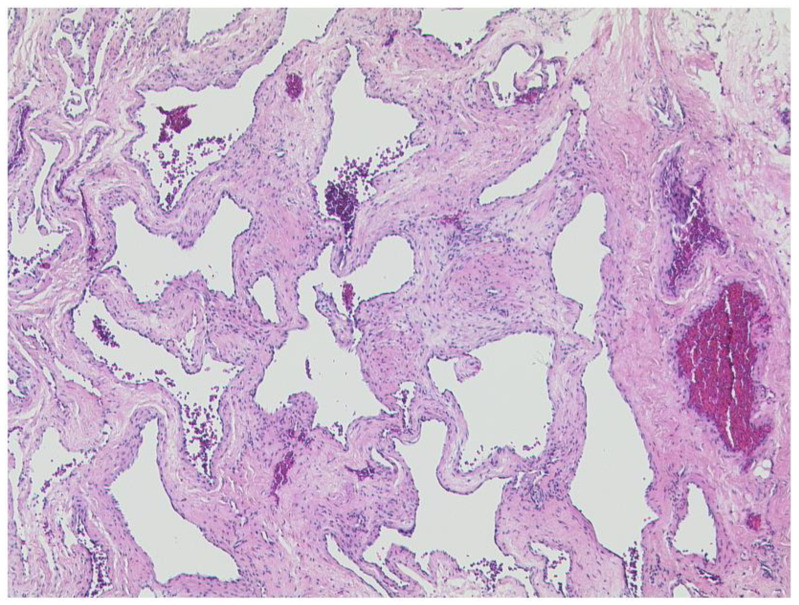
***Cavernous hemangioma.*** Abundant blood filled dilated vascular spaces with an inconspicious endothelial lining. HE, original magnification 50:1.

Bone remodeling or small calcifications can occur. Due to low vascular flow, mild contrast enhancement is typical. On MRI, cavernous hemangioma shows a heterogeneous signal on T1- and T2-weighted images, with mild contrast enhancement on T1-weighted images after contrast administration. Typically, an isointense signal compared to muscles can be found on T1-weighted images. Hyperintense signal can be found in case of thrombosis within the hemangioma. Flow voids within the mass are common [39,40]. Typical histopathology shows Figure 6.

Most of the cases are managed with a conservative therapy without surgery. Typical clinical classification systems are the **classification** of the ISSVA (International Society for the Study of Vascular Anomalies) and the Hamburg classification system of vascular malformations [41,42,43]. An overview is given on Table 3.

### 3.3. Extraconal Tumors

#### 3.3.1. Capillary Hemangioma

Capillary hemangioma is the most common benign tumor in infants [44]. It occurs five times more often in female than in male infants and appears as a raised, lumpy area of flesh in approximately 10% of all births [45,46]. After a proliferative phase of up to ten months, a slow involution phase for up to ten years follows [47].

The diagnosis is commonly made clinically, and the majority of patients do not require special treatment. Capillary hemangiomas are usually located anterior to the globe and are present at birth. When capillary hemangioma appears as a mass of the orbit and periorbital areas, imaging is often necessary to assess the extent of the hemangioma and its possible effect on adjacent anatomical structures [48]. Aside from the involvement of extraconal structures like extra-ocular muscles and lacrimal glands, intracranial extension through the optic canal or superior orbital fissure can also be found [49]. Clinically, superficial, deep, and mixed lesions can be differentiated. An overview of the patterns and different types is shown in Table 4.

Superficial lesions below the dermis tend to have a deep blue hue. These lesions can present as skin discoloration and can be seen from outside. In contrast to this, deep orbital lesions can be invisible from outside and could be detected by clinical symptoms of proptosis, strabismus, or decreased visual acuity. Mixed lesions show a combination of both manifestations [50].

**Figure 7:**
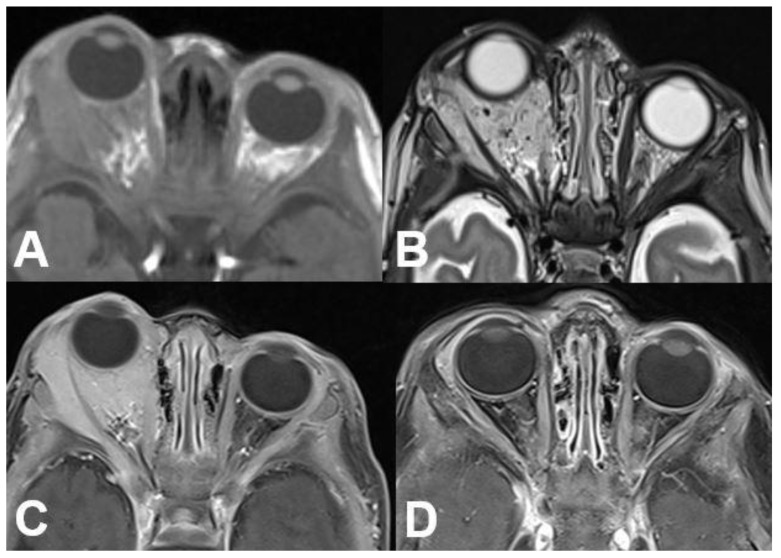
***Capillary hemangioma.*** MRI of a 12-week-old newborn female showing a tumor of the right orbit producing exophthalmos with slight isointense signal on T1-weighted images (**A**) compared to the orbit muscles and a hyperintense signal on T2-weighted images (**B**). The tumor shows homogenous enhancement in post-contrast images (**C**). After 11 months and therapy with propranolol, the tumor is not visible on exemplary post-contrast T1-weighted imaging (**D**).

On MRI with T-weighted images, capillary hemangioma appears as an extraconal, well-defined, lobular hyperintense mass (Figure 7). Septa or lobulated parts within the mass are common. Capillary hemangioma appears usually slightly hypointense on T1-weighted images, enhancement on T1-weighted imaging after contrast administration is typically intense and homogeneous with marked enhancement of intratumoral vessels. Fine internal flow voids can occur. Capillary hemangioma commonly respect the adjacent bony structures. True bone invasion or calcifications are very rare [51]. Differential diagnoses are other common vascular malformations of the orbit, such as cavernous hemangioma of the orbit or a venous varix.

**Figure 8:**
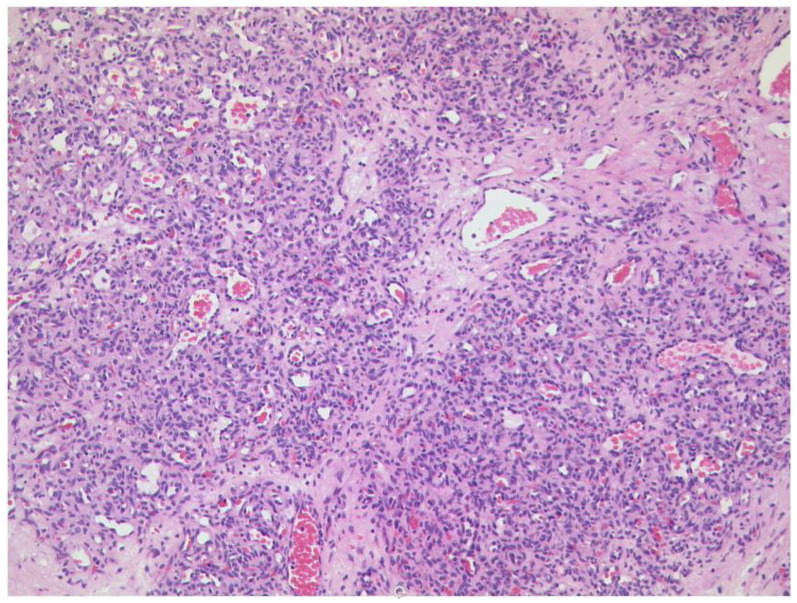
***Capillary hemangioma.*** Numerous small blood vessels with a lobulated architecture. The calibre of the vessels is distinctly and visibly smaller than in the cavernous hemangioma shown in Figure 6. HE, original magnification 100:1.

Figure 8 shows the histopathology of a capillary hemangioma of the anterior orbit/the skin of the eyelid.

#### 3.3.2. Lymphoma

Lymphoid orbital tumors, including benign reactive lymphoid processes and malignant lymphoma, represent 10 to 15% of all orbital tumors and 55% of ophthalmic malignancies [52]. Orbital lymphomas represent around 50 to 60% of ocular adnexal lymphoma [53].

**Figure 9:**
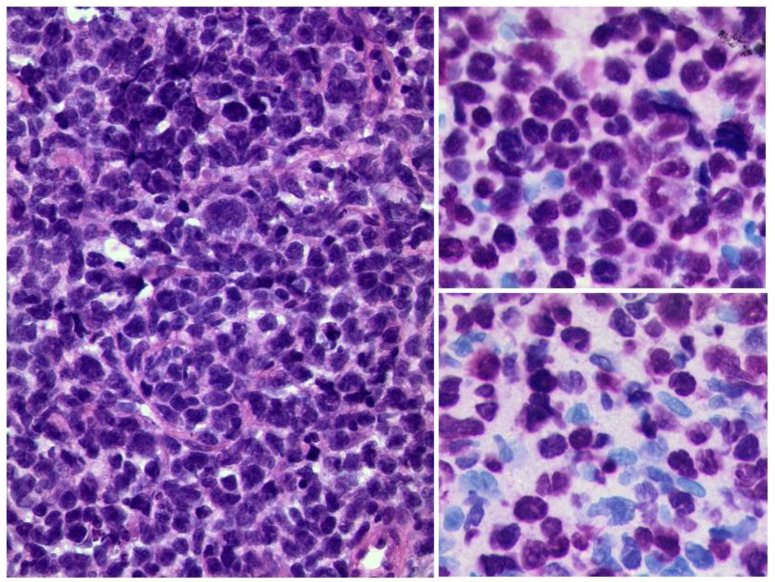
***Diffuse large B-cell lymphoma of the orbit.*** Left: diffuse infiltrate of blastic cells (HE, original magnification 400:1). The tumour cells are strongly positive for the B-cell marker Pax5 (upper right, original magnification 400:1) and have a high Ki-67 index of approximately 80% (lower right, original magnification 400:1).

Malignant orbital lymphoma typically arises from the mucosa-associated lymphoid tissue of ocular adnexa as a B-cell non-Hodgkin lymphoma. In addition, in 1.5 to 5% of patients with systemic lymphoma, the orbit can be involved [54]. In more than 80% of the cases, diffuse large B-cell lymphoma (DLBCL) is the underlying subtype (Figure 9).

**Figure 10:**
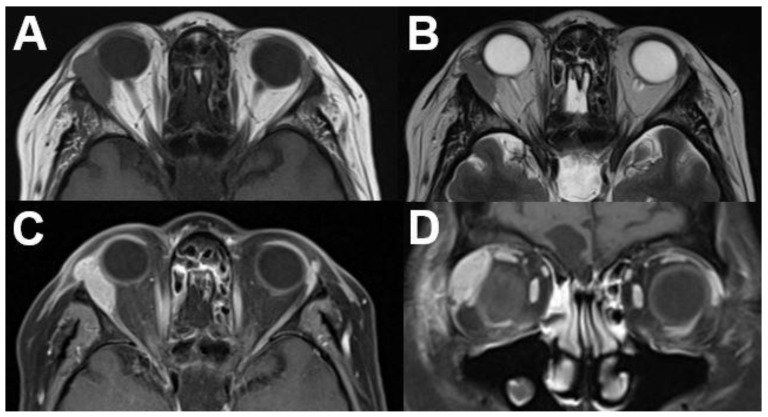
***Mucosa-associated lymphoid tissue (MALT) lymphoma.*** MRI of a 54-year-old female with a right-sided primary orbital MALT lymphoma of the lacrimal gland. T1-weighted (**A**) and T2-weighted (**B**) images show a well-defined lobulated tumor with homogenous contrast enhancement on axial (**C**) and coronal view (**D**). Infiltration of adjacent structures cannot be identified.

Most patients are elderly and between 50 and 70 years of age. No gender predilection is known in the current literature; however, this varies with the histological subtype [6]. Lymphoma can involve every part of the orbit, the superolateral orbit is most often affected and bilateral appearance is common [55]. The vast majority of B-cell lymphomas (90%) present as a unilateral tumor, e.g., mucosa-associated lymphoid tissue (MALT) lymphomas (Figure 10).

**Figure 11:**
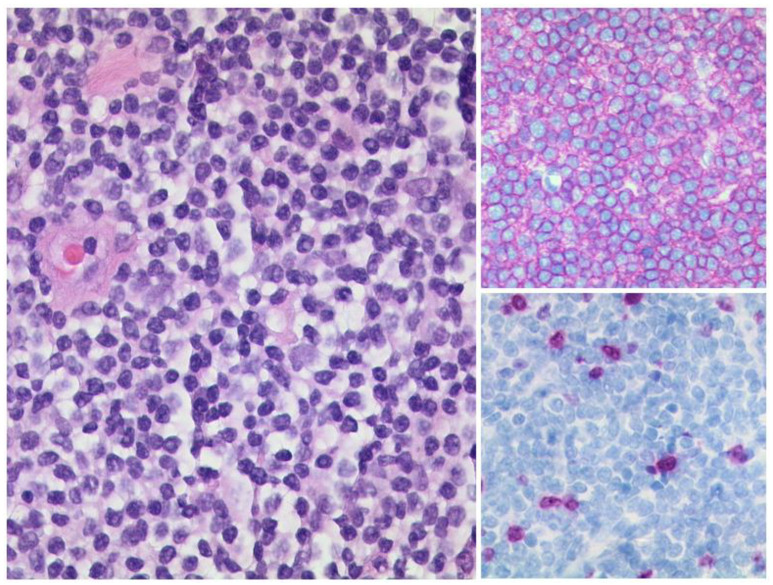
***Mucosa-associated lymphoid tissue (MALT) lymphoma.*** The lymphoma consists of small, relatively uniform lymphocytes (left, HE, original magnification 400:1). The lymphoma cells show a strong and uniform expression of CD20 (upper right, original magnification 400:1) and have a low proliferation rate (lower right, Ki-67-staining, original magnification 400:1).

The corresponding histopathology is illustrated in Figure 11.

**Figure 12:**
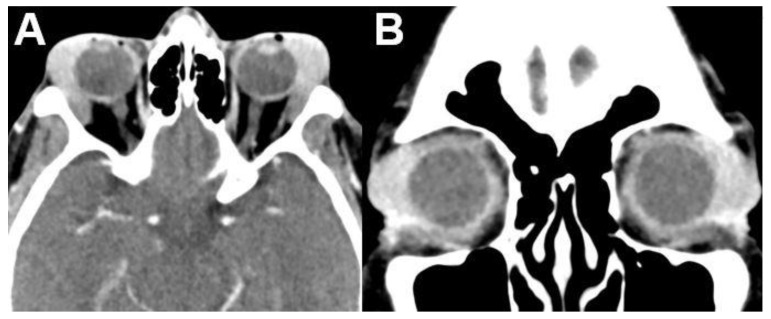
***Mantle cell lymphoma.*** CT of a 60–year-old female patient with mantle cell lymphoma showing bilateral involvement of the lacrimal gland. On axial (**A**) and coronal (**B**) images, lymphoma appears as a well-defined, homogenously enhancing mass of the bilateral lacrimal glands.

However, especially in mantle cell lymphoma, a bilateral tumor can be registered [53] (Figure 12).

On CT and MRI, lymphoma appears as a well-circumscribed, homogenous, typically lobulated mass. Tumors mold around normal anatomic orbital structures. Typically, they do not erode surrounding bony structures. On CT, lymphoma appears isodense to skeletal muscles with homogenous contrast enhancement. A homogenous intermediate signal on T1- and T2-weighted imaging, with homogenous contrast enhancement on T1-weighted imaging after contrast administration, is typical. The signal on T1-weighted images is usually iso- to hypointense compared to muscle signal, reflecting a high cell density. Signal on T2-weighted images is usually hyperintense compared to muscle signal but can be hypointense to the signal of the bulbus or cerebrospinal fluid. Due to its hypercellular nature, high signal on DWI and signal loss on ADC maps can help to distinguish lymphomas from inflammation. An affection of the lacrimal gland is possible.

#### 3.3.3. Tumors of the Lacrimal Gland

Lacrimal gland tumors are rare and account for about 10% of orbital masses. These tumors include a wide spectrum ranging from benign epithelial and lymphoid lesions to carcinomas, also including lymphomas and sarcomas with respective differences in the choice of treatment and related prognosis [56]. An overview of different tumors of the lacrimal gland is shown in Table 5.

However, for definite diagnosis, excision or biopsy is required, and radiological imaging can help to distinguish between benign conditions (well defined, oval masses) or malignant masses (invasion of adjacent structures, bone destruction). Furthermore, CT or MRI findings can be used to plan surgical excision/biopsy and further treatment, e.g., chemotherapy. Pleomorphic adenoma is the most common benign epithelial tumor. On CT images a typically well defined, unilateral tumor with moderate contrast enhancement that involves the orbital lobe of the lacrimal gland can be seen. On MRI pleomorphic adenoma present with low to intermediate T1 signal and intermediate T2 signal compared with orbit muscles. After contrast admission they show moderate enhancement [57].

**Figure 13:**
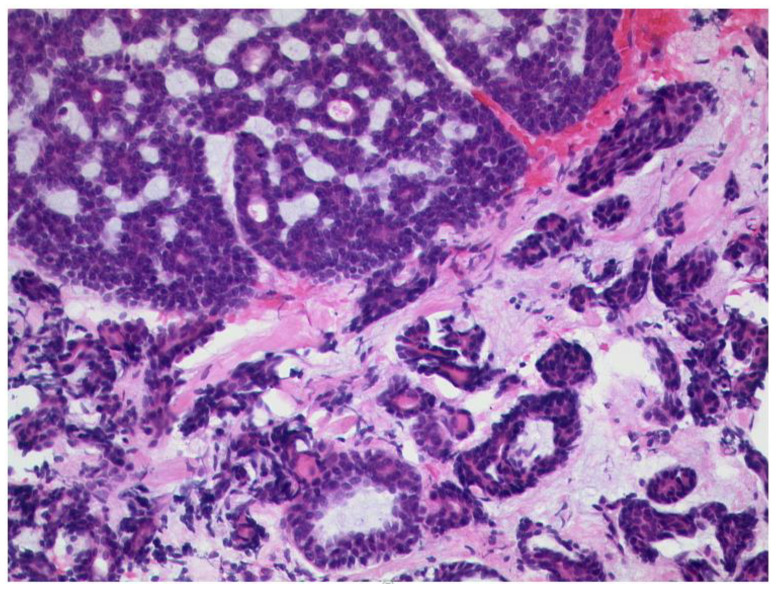
***Adenoid cystic carcinoma of the lacrimalgland.*** Classical morphology with a cribriform pattern (upper left) and a coexisting tubular pattern (lower half of the picture), HE, original magnification 200:1.

**Figure 14:**
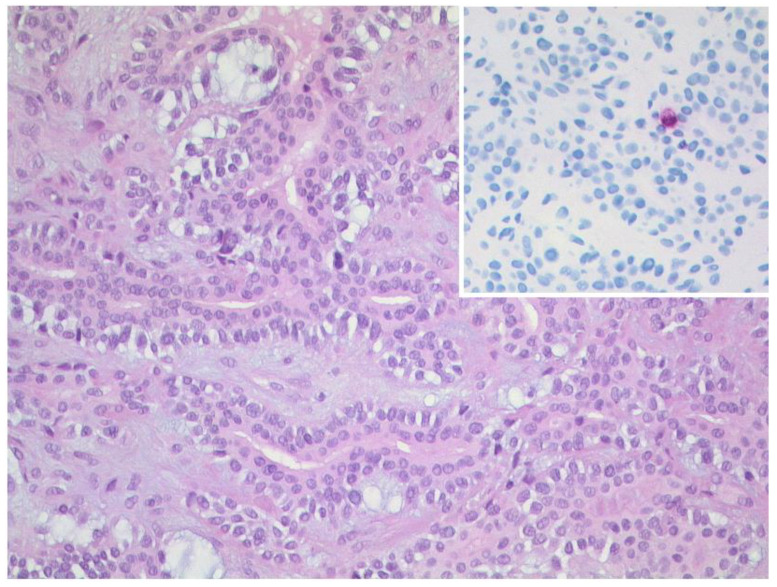
***Pleomorphic adenoma of the lacrimal gland.*** The tumor shows epithelial, myoepithelial, and stromal elements (HE, original magnification 200:1). Inset: The proliferation rate is very low, Ki-67 index < 1% (original magnification 200:1).

The most common epithelial tumors are benign pleomorphic adenoma. Among lacrimal gland masses, malignant epithelial tumors account for approximately 5% of all orbital lesions. The most common malignant lacrimal gland tumors are adenoid cystic carcinomas and carcinomas (Figure 13 and Figure 14).

**Figure 15:**
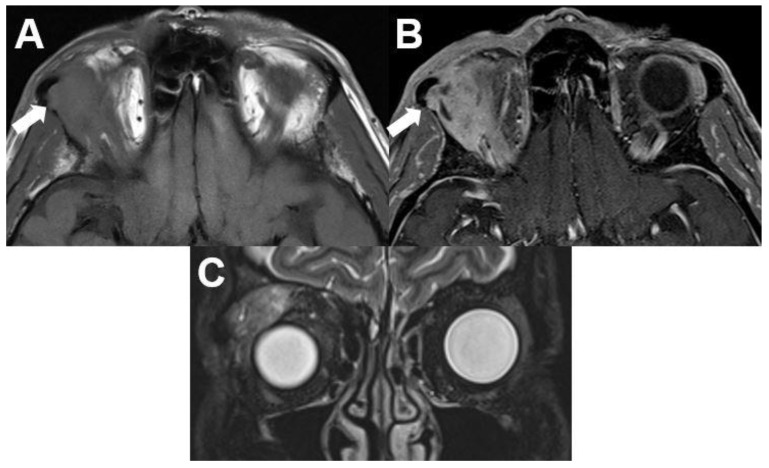
***Carcinoma of the lacrimal gland.*** MRI of a 59-year-old male patient with a biopsy-proven poorly differentiated carcinoma of the right lacrimal gland. T1-weighted axial images show an ill-defined mass of the right lateral cranial orbit with signal characteristics similar to surrounding muscles (**A**) and contrast enhancement (**B**). The adjacent zygomatic bone is infiltrated (arrow). On T2-weighted images of the coronal view, the tumor shows an inhomogeneous signal (**C**).

Patients present with symptoms such as pseudoptosis, exophthalmos, dystopia, pain, and reduced visual acuity [58]. On CT, adenoid cystic carcinoma presents as a homogenous, well-defined tumor with diffuse contrast enhancement and possible calcifications. The destruction of adjacent bone is also typical. MRI shows the hypointense T1 signal and intermediate to hyperintense signal on T2-weighted images. After contrast admission adenoid cystic carcinoma also appears with diffuse enhancement [59,60]. Compared with adenoid cystic carcinoma, carcinomas arising from primary pleomorphic adenomas, adenocarcinomas, and mucoepidermoid carcinomas are less common [61,62]. Figure 15 shows exemplary MRI of a patient with carcinoma of the right lacrimal gland.

### 3.4. N. opticus

#### 3.4.1. Optic Nerve Glioma

As a slow growing tumor, optic nerve glioma occurs typically in children. In 12 to 37% of cases, it appears in patients with neurofibromatosis type 1 (NF1), often with bilateral manifestation. In patients with NF1, it is associated with a better prognosis compared to other cases [63]. Optic nerve glioma most often appears in the first decade with a peak incidence between 2 to 8 years [64]. The vast majority of patients are affected in the first two decades of life [65]. Optic nerve glioma makes 1% of orbital tumors and up to 7.0% of gliomas [66]. Patients typically show slowly progressive visual loss, nystagmus, visual field defects, and afferent pupillary defects, followed by proptosis at later stages [64]. Optic nerve glioma regularly shows periods of growth and dormancy [67]. However, any part of the optic nerve may be involved and almost half of all gliomas are located within the optical nerve. Another 10% involve the intracranial optic nerve and chiasm and up to 5% only affect the chiasm [67]. Malignant gliomas are rare and typically affect adult male patients. Patients show a very poor prognosis, as the majority of patients commonly die within one year [68]. Thr initial symptoms of malignant gliomas include retroorbital pain, progressive uni- or bilateral visual loss, and the swelling/hemorrhaging of the optic nerve head [69].

**Figure 16:**
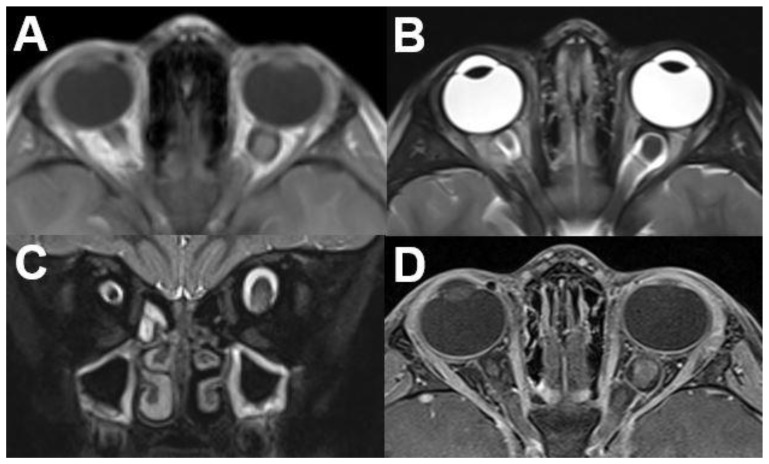
***Neurofibromatosis type I (NF1).*** MRI of a 5-year-old female with NF1. The left optic nerve is thickened on axial T1-weighted (**A**) and T2-weighted images in axial (**B**) and coronal view (**C**). Additionally, a slight contrast enhancement can be detected (**D**).

On the CT, a widening of the optic canal can be seen in the bone window. Furthermore, MRI typically shows a well-defined, fusiform enlargement and tortuosity of the optic nerve [65]. A comparison to the contralateral side can help to distinguish the tumor (Figure 16).

On T1-weighted sequences the tumor shows a hypo- or slight hyperintense appearance compared to the contralateral side. T2-weighted images show a hyperintense mass as compared to the cerebral cortex. Sometimes cystic degeneration may occur. Additionally, a slight contrast enhancement or sometimes no enhancement can be seen on T1-weighted imaging after contrast administration. Optic nerve gliomas with the involvement of the intraorbital nerve and the chiasm can show the shape of a dumbbell.

**Figure 17:**
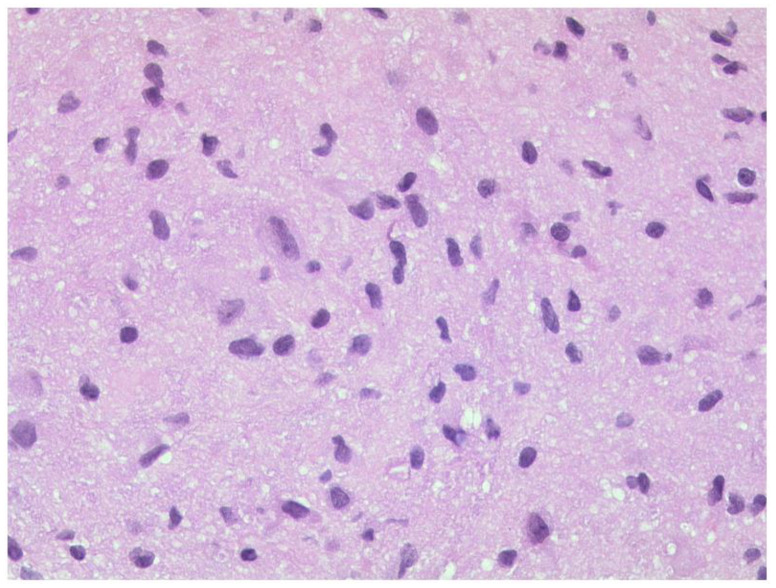
***Optic nerve glioma.*** A pilocytic astrocytoma with moderately anisokaryotic, slightly elongated nuclei embedded in a fibrillary background. HE, original magnification 400:1.

T1-weighted images without fat saturation are suitable for measuring the size and for delineating the course of the optic nerve. The normal maximum diameter in children is 5 mm or less. Hemorrhage or calcification are rare [65]. The involvement of the intracanalicular and retrocanalicular portions of the optic nerve has important implications regarding treatment and should be mentioned in the radiological report [65]. Further signs of intracranial NF should be specifically sought after. An example of the histopathological features of optic nerve glioma is illustrated in Figure 17.

#### 3.4.2. Optic Nerve Sheath Meningioma

The optic nerve sheath meningioma (ONSM) accounts for 1 to 2% of all meningiomas. In addition, it is the second most frequent primary optic nerve tumor [70]. ONSM commonly occurs in adult females in their fourth or fifth decade of life. The incidence in women is three times higher than in men [71]. ONSM typically presents unilaterally; however, in patients with NF type 2 (NF2) bilateral presentation may occur [70,72]. Patients complain about painless, slowly worsening loss of vision and proptosis, together with optic atrophy and optociliary shunt vessels. Sometimes extraocular motility deficits can also be present. Primary meningioma arises from capillary cells of the arachnoid around the intraorbital/intracanalicular part of the optic nerve. Secondary meningioma occurs intracranially from the sphenoid ridge, olfactory groove, or tuberculum sellae with an extension between the dura and arachnoid of the optic nerve invading the optic canal and orbit [70].

On MRI, ONSM typically shows intermediate and homogenous signal on T1- and T2-weighted images. On T1-weighted imaging after contrast administration, ONSM appears as a homogenously enhancing tumor surrounding the optic nerve. CT may show calcification within the lesion. The optic nerve can be located in the center or eccentrically in relation to the mass. Post-contrast T1-weighted, fat-suppressed images are ideal for the diagnosis. Sometimes a characteristic “tram track sign” on axial images or a “doughnut sign” on coronal images can be detected [4].

Meningiomas tend to cause the segmental or diffuse circumferential thickening of the optic nerve sheath so that on sagittal images this produces a tram track sign [73]. The extent of ONSM ranges between a thin sheet around the optic nerve to an expansive mass. For the differentiation of ONSM from optic glioma it is important to consider that glioma expands the optic nerve. ONSM can be distinguished from lymphoma by the effect that lymphoma shows less intense enhancement. In addition, untreated lymphoma shows no calcifications. Appearances to distinguish between optic nerve glioma and ONSM on CT and MRI are described in Table 6.

**Figure 18:**
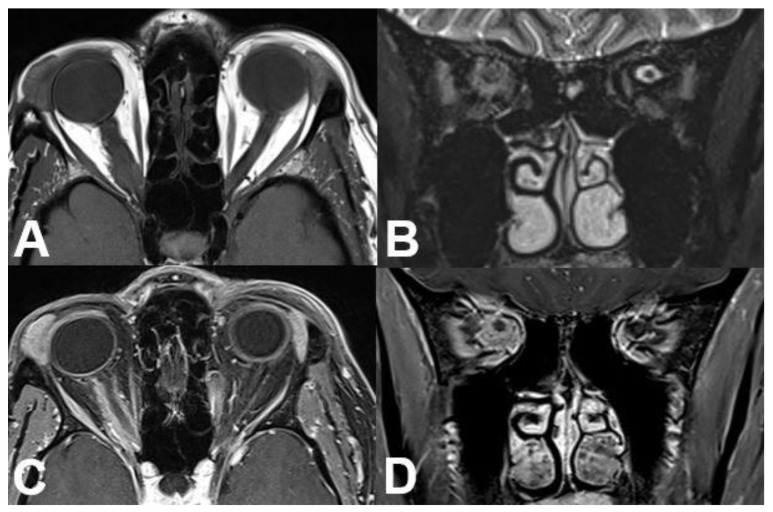
***Optic nerve sheath meningioma (ONSM).*** MRI of a 43-year-old female patient with a well-defined mass around the optic nerve with T1-hypointense (**A**) and T2-hyperintense signal (**B**). After contrast administration (**C**,**D**), a homogenous enhancement is seen with sparing of the optic nerve (“tram track sign”).

**Figure 19:**
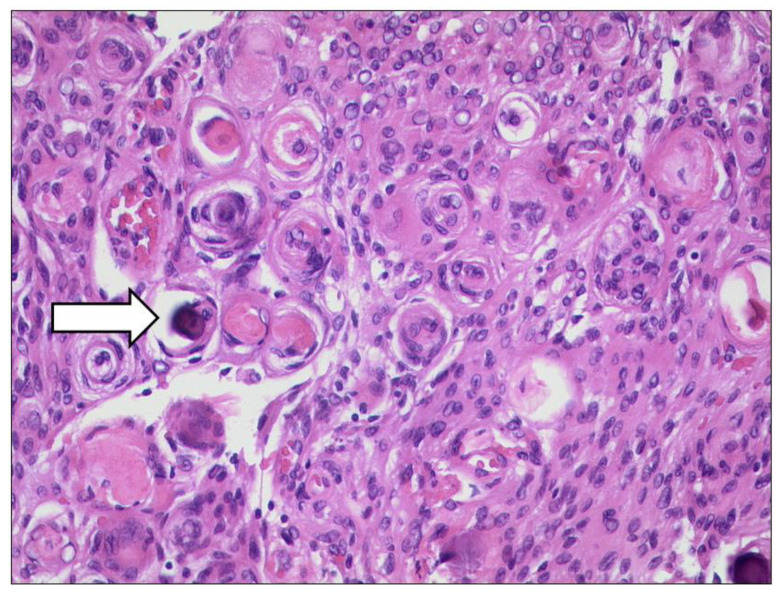
***Optic nerve sheath meningioma (ONSM).*** The tumour cells build many whorls and there are interspersed psammoma bodies (arrow). (HE, original magnification 200:1).

An example of a patient with ONSM is shown in Figure 18 with typical histopathology in Figure 19.

### 3.5. Peripheral Nerve Sheath

#### 3.5.1. Schwannoma

Schwannomas are the most common benign peripheral nerve tumors in adults. Any nerve with Schwann cells in the perineurium can be affected, including all cranial nerves (except for optic and olfactory nerve), spinal nerves, and the autonomic nervous system [74]. Generally, schwannomas are benign, slow growing nerve sheath tumors, originating from the Schwann cells of the perineurium of peripheral nerves [75]. Of all schwannomas, 25 to 45% occur in the head and neck; orbital schwannomas account for only 1% of all orbital tumors [76]. However, while they can present at any age, patients are typically between their third and sixth decades of life [77]. Most commonly, orbital schwannomas present with painless, non-pulsatile ocular proptosis, the displacement of the globe, or a palpable mass in the orbit. Lid swelling is also possible [78,79]. Due to the complexity of nerve fibers in the orbit, the specific nerve of origin can often not be identified. Most commonly, orbital schwannomas arise from the supraorbital and supratrochlear sensory nerves and are therefore primarily located in the supraorbital region [80].

**Figure 20:**
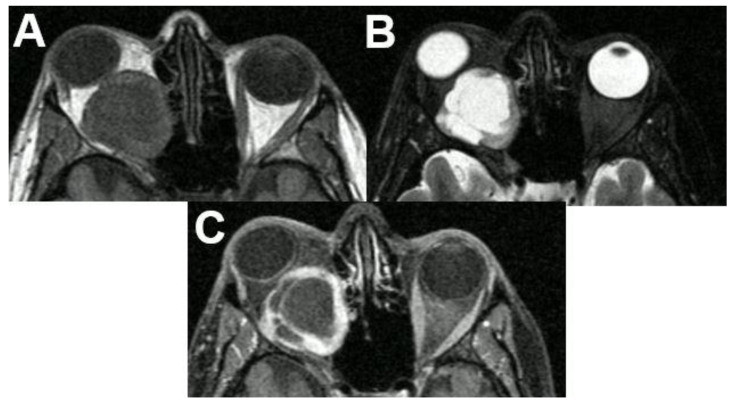
***Schwannoma of the optic nerve.*** MRI of a 36-year-old female presenting with a large retrobulbar tumor and exophthalmos. The tumor is hypointense on T1-weighted (**A**), hyperintense on T2-weighted sequences (**B**), and shows avid contrast enhancement of its solid parts (**C**). Note the cystic degeneration of the tumor. The tumor was surgically removed, and pathology revealed a schwannoma.

It is often difficult to differentiate schwannomas clinically from other orbital tumors. Typical imaging findings can help to predict the diagnosis of a schwannoma. On MRI, orbital schwannoma presents as a lobular, heterogeneously enhancing mass on T1-weighted imaging after contrast administration, with isointense or hypointense signal on non-contrast T1-weighted and marked hyperintense signal on T2-weighted sequences (Figure 20).

**Figure 21:**
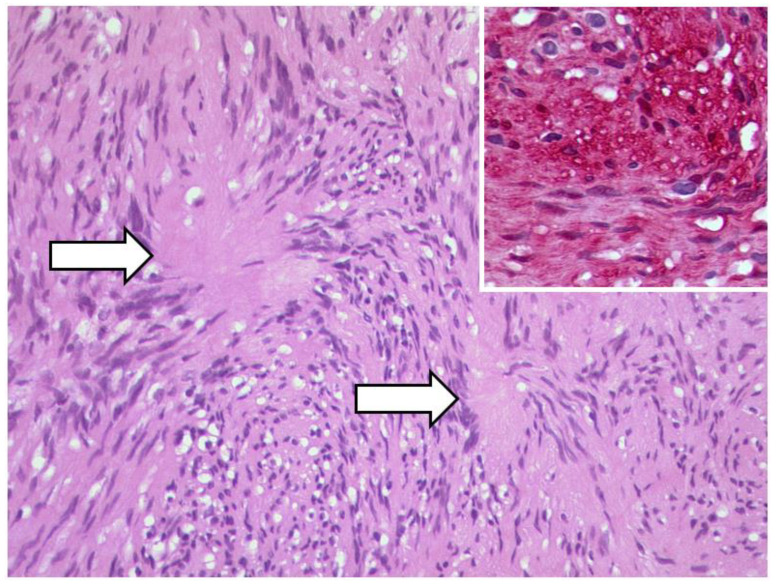
***Schwannoma of the optic nerve.*** Antoni A pattern and verrocay bodies (arrows) of a schwannoma, HE, original magnification 200:1. Inset: the tumor cells show a strong and diffuse expression of S100 (original magnification 200:1).

Contrast enhancement can either be distributed homogenously or heterogeneously [75,76,80]. Sometimes differentiation between schwannoma and cavernous hemangioma can be challenging. Thus, it has been proposed that the contrast enhancement spread pattern using dynamic MRI can be helpful for this differentiation: while hemangiomas present with initial enhancement from one specific point or portion, schwannomas show enhancement from a wider area [81]. Moreover, CT is helpful for assessing bony expansion and for surgical planning. On CT imaging, orbital schwannomas are smooth, elongated, and have an oval to spindle shape, with a density similar to extraocular muscle. CT may show characteristic expansion into bone, without bony erosion [75]. Typical histopathology of a schwannoma of the optic nerve shows Figure 21.

#### 3.5.2. Neurofibroma

Peripheral neurofibromas are benign peripheral nerve sheath tumors composed of Schwann cells, fibroblasts, perineurial cells, and mast cells [82]. They can manifest within the orbit, and they can be associated with systemic NF [83]. Neurofibromas are uncommon orbital lesions, accounting for approximately 2 to 4% of orbital tumors [82]. The three general types of neurofibroma are cutaneous, nodular, and plexiform. Plexiform neurofibromas are less common than the other forms. They can involve the orbit and are associated with increased mortality in patients with NF1 because of possible malignant transformation. In approximately 10% of NF1 patients, plexiform neurofibromas transform into malignant peripheral nerve sheath tumors [84]. Although cases of orbital plexiform neurofibroma are reported without other typical findings of NF1, it is highly suggestive for the diagnosis [85]. Orbital neurofibromas typically present with the progressive symptoms of an orbital mass, including proptosis, globe displacement, impaired extraocular motility, ptosis, and sensory dysfunction. Plexiform neurofibromas often manifest during the first decade of life, whereas localized neurofibromas are present in the third to fifth decade [83].

**Figure 22:**
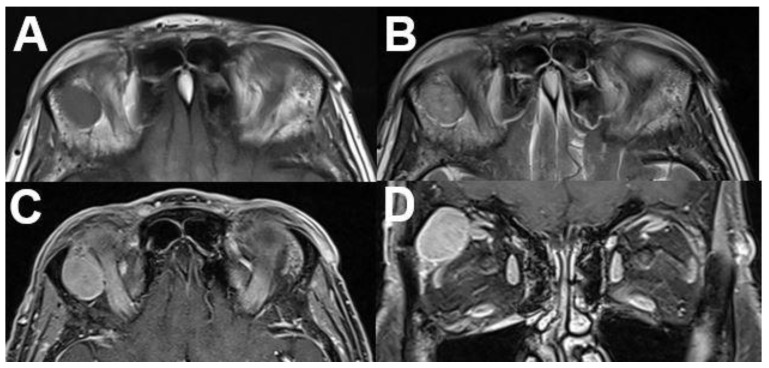
***Neurofibroma.*** MRI of a 59-year-old female patient with a tumor of the right-sided upper orbit. The tumor is located between the superior and lateral rectus muscle, showing T1-isointense (**A**) and slight T2-hyperintense (**B**) signal compared to the muscles. It shows heterogeneous contrast enhancement on axial (**C**) and coronal (**D**) views. In this case, diagnosis was proven after resection.

**Figure 23:**
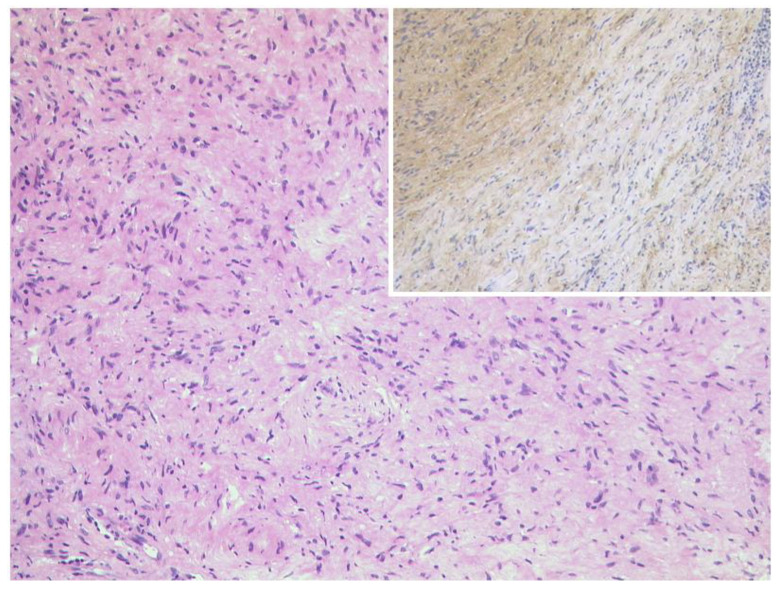
***Neurofibroma.*** Left: Spindle cell tumour with wavy nuclei and eosinophilic cytoplasm. No nuclear atypia and no mitosis are present. HE, original magnification 100:1. The expression of S100 is weaker and less consistent than in schwannoma, original magnification 100:1.

On cross-sectional imaging plexiform neurofibromas may involve large portions of the face in the form of a serpentine soft tissue mass. On T1-weighted images, they present with a heterogeneous signal, and on T2-weighted images with a hyperintense signal. The tumor typically shows heterogeneous contrast enhancement on T1-weighted sequences after contrast administration [2,86]. Localized neurofibromas present as smoothly marginated ovoid lesions that may be lobulated. On CT, they appear isodense or hypodense compared to extraocular muscles and show variable contrast enhancement with possible ring-enhancing characteristics. On MRI, low to moderate signal on T1-weighted and moderate to high signal on T2-weighted imaging can be observed for localized neurofibromas. There may be a heterogeneity of signal strength within the lesion. Contrast enhancement is variable, either on CT and MRI [87]. An example is illustrated in Figure 22 with the corresponding histopathology in Figure 23.

### 3.6. Masses with Involvement of Different Parts of the Orbit

#### 3.6.1. Metastasis

Orbital metastases are reported in the literature with an incidence of up to 13% of all orbital tumors [88]. In 2 to 5% of patients with systemic cancer, orbital metastases are reported; however, orbital involvement commonly presents late in the course of a multi-systemic disease. Nevertheless, isolated cases are reported, in which orbital metastasis is the first sign for abdominal or thoracic primary malignancies [89]. The most common primary cancer manifestations with orbital metastases are breast, lung, and prostate cancer; however, breast cancer is leading [88]. Table 7 gives an overview of the most common origins of orbital metastases.

The mean age is over 60 years when orbital metastatic masses are diagnosed [90]. Clinical symptoms are diplopia (48%), pain (42%), visual loss (30%), or proptosis (up to 63%) but strabismus or palpable masses are also reported [91]. Overall, the incidence of reported orbital metastases has been increasing due to improved tumor treatments and wide availability of CT and MRI.

**Figure 24:**
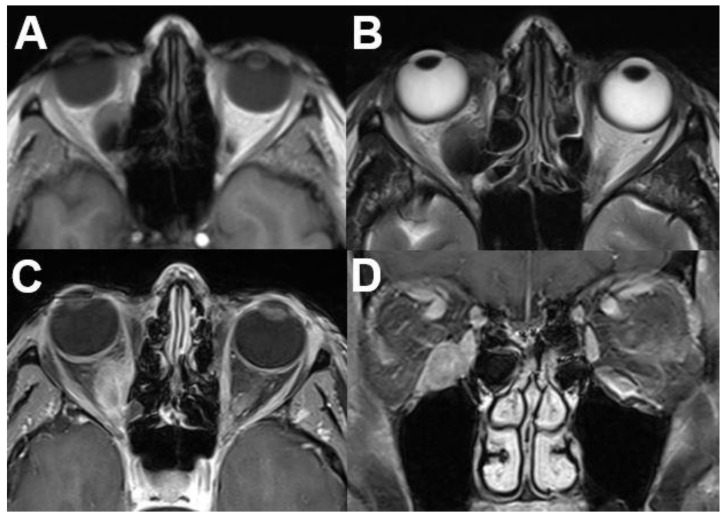
***Metastasis.*** MRI of a 56-year-old female patient with breast cancer. MRI shows an ill-defined tumor in the right-sided medial orbit showing T1-hypointense (**A**) and T2-hypointense (**B**) signal and inhomogeneous contrast enhancement (**C**,**D**). The tumor is in contact with the optic nerve and infiltrates the medial and inferior rectus muscle.

**Figure 25:**
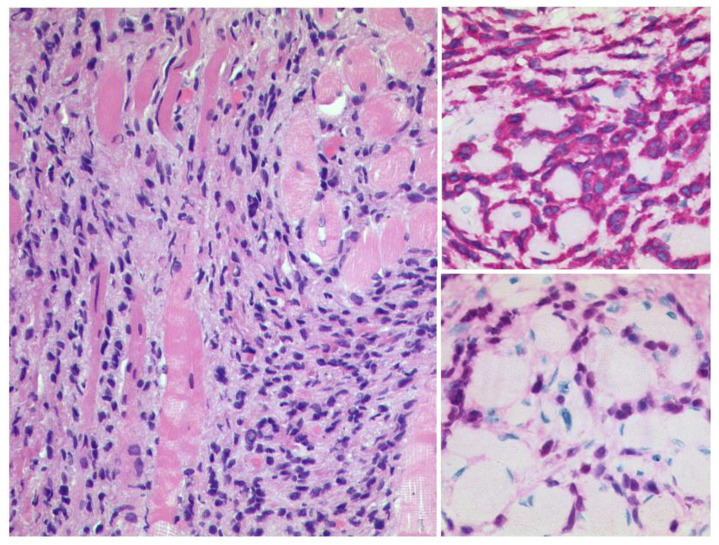
***Metastasis of a lobular breast carcinoma.*** Left: The carcinoma cells infiltrate diffusely between the sceletal muscle fibers of the extraocular muscles, HE, orifinal magnification 200:1. The carcinoma cells express strongly CK7 (upper right, original magnification 400:1) and estrogen receptor (lower right, original magnification 400:1).

A lesion without any symptoms and empty tumor anamnesis suggests a benign lesion. In case of missing anamnesis, a biopsy by fine-needle aspiration is preferred over an open biopsy. Metastatic lesions are more common in the anterior orbit than posterior orbit [92]; however, every part of the orbit can be affected. Signs of muscle infiltration or bone erosion always suggests malign genesis. Metastases from renal cell carcinoma and melanoma tend to be more circumscribed than those stemming from other primary cancer entities [88]. All orbital metastases show enhancement on T1-weighted imaging after contrast administration, a low signal on non-contrast T1-weighted images and intermediate to high signal intensity on T2-weighted images are common. An example of a patient with orbital metastasis is shown in Figure 24 and Figure 25.

**Table 7:** Most common origins of orbital metastasis according to *Shields*, *Carol L.* et al. *“Cancer Metastatic to the Orbit.” (2001)* [92].

Primary Site	Tumor Type	%
Breast	Carcinoma	53
Prostate gland	Carcinoma	12
Lung	Carcinoma	8
Skin (melanoma)	Melanoma	6
Kidney	Carcinoma/Sarcoma	5
Alimentary tract	Carcinoma/Carcinoid	5

#### 3.6.2. Rhabdomyosarcoma

**Figure 26:**
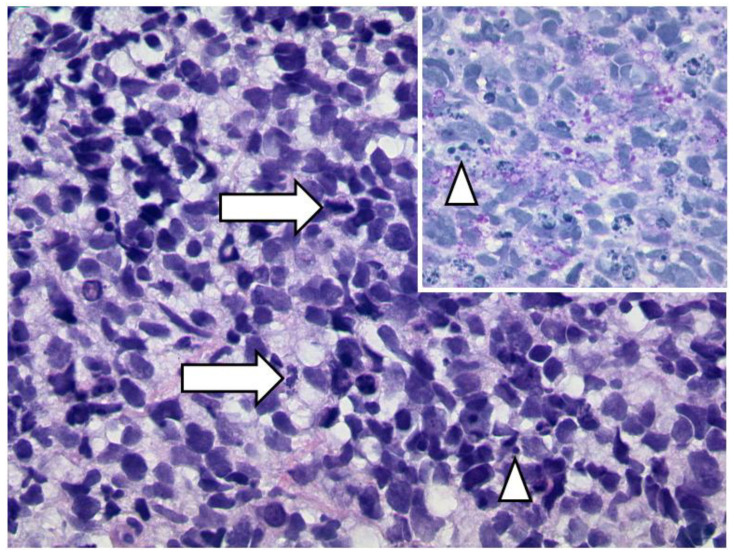
***Embryonal rhabdomyosarcoma of the orbit.*** A diffuse infiltrate of clearly malignant blastic cells with many mitotic figures (arrows) and abundant apoptotic figures (arrowheads) (HE, original magnification 400:1). The cytoplasm contains glycogen granula (inset, PAS-stain, original magnification 400:1).

Rhabdomyosarcoma (RMS) is a very rare malignant tumor of the childhood. Approximately 90% of orbital RMS occur before the age of 16 years [93]. The head and neck region and especially the orbit is often involved in RMS, with 10% of all RMS cases present with a primary localization in the orbit (76%). The orbit as the primary localization is followed by the conjunctiva (12%), uveal tract (9%), and the eyelid (3%). In addition, RMS originating from the nasopharynx or the paranasal sinuses can involve the orbit. A secondary ophthalmic metastasis from distant RMS is very rare [92,94,95]. Usually, RMS clinically appears as a rapidly growing mass. Histopathologically, three subtypes can be divided: embryonal, alveolar, and mixed. Figure 26 shows an example of an embryonal rhabdomyosarcoma of the orbit.

**Figure 27:**
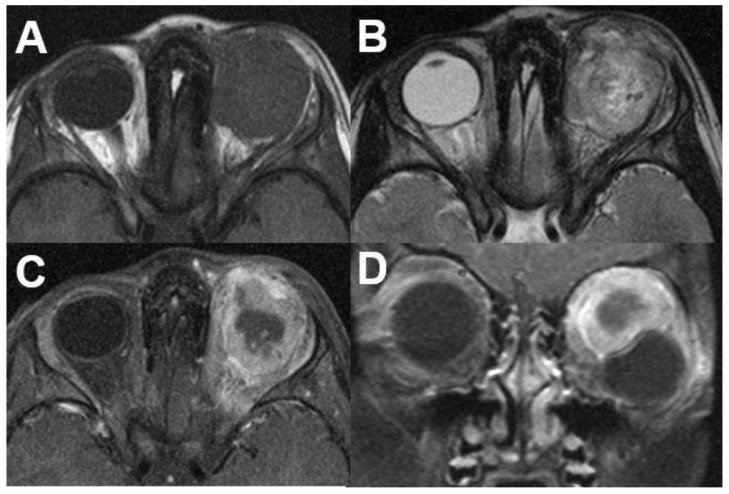
***Rhabdomyosarcoma (RMS).*** MRI of a 4-year-old female patient with RMS of the left-sided orbit. The tumor shows hypointense signal on T1-weighted images (**A**) and inhomogeneous hyperintense signal on T2-weighted images (**B**). The center of the lesion shows marked hyperintensity compared to the contralateral side. Peripheral enhancement and central absent enhancement is seen after contrast administration, matching necrotic portions (**C**). The bulb is compressed and displaced caudally (**D**).

For the diagnosis and grading of RMS, a biopsy is the reference standard [96]. CT and MRI are important for initial staging and further treatment. On CT and MRI, RMS appears as a well-defined, homogenous mass. On CT images, it is isodense compared to the extraocular muscles with contrast enhancement. On T1-weighted images, RMS demonstrates an isointense signal compared to extraocular muscles and hypointense signal compared to orbital fat. On T2-weighted images, it is hyperintense to extraocular muscles and orbital fat. Bony erosion and infiltration into the paranasal sinuses and nasopharynx are possible. Areas of hemorrhage are rare but possible, especially in alveolar and pleomorphic subtypes. An example of a patient with RMS is shown in Figure 27.

## 4. Conclusions

Benign and malignant orbital tumors can be challenging for image interpretation. Apart from their typical clinical presentation, the radiologist should know characteristic appearances and key features on cross-sectional images (CT an MRI) to determine the correct diagnosis and distinguish between similar entities. Moreover, the precise description of lesion location, involved orbital compartments, and the extent is crucial for further treatment. This pictorial review should help the radiologist in the diagnosis and evaluation of orbital tumors.

## Figures and Tables

**Table 1:** Exemplary sequence protocol for orbit MRI. TSE: turbo spin echo; TIRM: turbo inversion recovery magnitude; VIBE: volume interpolated breath-hold examination; DWI: diffusion weighted imaging; ADC: apparent diffusion coefficient.

Signal	Sequence	Plane	Slice Thickness	Description
T1 weighted	T1-TSE	axial	3 mm	
T2-weighted	T2-TSE	axial	3 mm	
TIRM		coronal	4 mm	from the orbit to the chiasm
Post-contrast	T1-VIBE fat-saturated	axial and coronal, sagittal (optional)	axial 1 mm coronal 2 mm sagittal 3 mm	axial for the orbits, coronal from the orbit to the chiasm
DWI	b = 0; b = 1000; ADC-map	axial	3 mm	

**Table 2:** Grouping System according to the International Classification of Retinoblastoma.

A	Small	− 3 mm in any diameter − located >3 mm from the foveola − located >1.5 mm from the optic disc
B	Large	macular or juxtapapillary location
C	Local Dissemination	discrete vitreous or subretinal seeding
D	Diffuse	− tumor is massive − diffuse vitreous seeding − subretinal seeds
E	Unsalvageable or extensive	− neovascular glaucoma − intraocular or corneal hemorrhage − tumor contact with the lens − tumor in the anterior segment − diffuse infiltrating retinoblastoma
F	Extrascleral	extrascleral spread (optic nerve, orbit or distant metastases)

**Table 3:** Overview from the classification for vascular anomalies (ISSVA).

Vascular Anomalies				
Vascular Tumors	Vascular Malformation			
	Simple	Combined	of Major Named Vessels	Associated with Other Anomalies
Benign	Capillary malformations	CVM, CLM		
Local aggressive or borderline	Lymphatic malformations	LVM, CLVM		
	Venous malformations	CAVM		
Malignant	Arteriovenous malformations	CLAVM		
	Arteriovenous fistula	others		

**Table 4:** Infant hemangioma according to the classification for vascular anomalies (ISSVA).

Pattern	Different Types
focal	Superficial
multifocal	Deep
segmental	Mixed (superficial + deep)
indeterminate	Reticular/abortive/minimal growth
	others

**Table 5:** Benign and neoplastic lacrimal gland tumors.

Inflammatory	Neoplastic		
Sarcoidosis	Benign epithelial tumors	Malignant epithelial tumors	Non-epithelial neoplasmas
Orbital inflammatory pseudotumor	Pleomorphic adenoma	Adenoid cystic carcinoma	Lymphoma
Sjögren syndrome	Oncocytoma	Adenocarcinoma	Chloroma
Infectous dacryoadenitis	Dermoid cyst	Acinic cell carcinoma	Haemangioma
		Squamous cell carcinoma	Metastatic or secondary tumors

**Table 6:** Radiological characteristics of optic nerve sheath meningiomas and optic nerve gliomas https://casebasedneuroophthalmology.pressbooks.com/chapter/optic-nerve-sheath-meningioma/ (accessed on 17 August 2022).

Optic Nerve Meningioma	Optic Nerve Glioma
Calcification of the optic nerve sheath and adjacent bony hyperostosis on CT	No calcification or hyperostosis on CT
Apical expansion of the tumor	Kinking or buckling of the optic nerve
Optical nerve sheath thickening and enhancement with relative sparing of the optic nerve substance (tram tack sign)	Thickening of optic nerve and sheath by the tumor
Prominent contrast enhancement on MRI and CT	Variable contrast enhancement on MRI and CT
Extradural tumor extension	Cystic spaces within the optic nerve

## Data Availability

Not applicable.
